# Discontinuation and reinitiation of pharmacological treatment for ADHD among individuals with ADHD and substance use disorder

**DOI:** 10.1136/bmjment-2025-302138

**Published:** 2026-03-23

**Authors:** Andrea J Capusan, Le Zhang, Henrik Larsson, Isabell Brikell, Diana Martinez, Brian M D’Onofrio, Paul Lichtenstein, Ralf Kuja-Halkola, Zheng Chang

**Affiliations:** 1 Center for Social and Affective Neuroscience, Department of Biomedical and Clinical Sciences, Linköping University, Linköping, Sweden; 2 School of Public Health, Shenzhen University, Shenzhen, Guangzhou, People's Republic of China; 3 Department of Medical Epidemiology and Biostatistics, Karolinska Institutet, Stockholm, Sweden; 4 School of Medical Sciences, Örebro University, Örebro, Sweden; 5 Department of Biomedicine, Aarhus University, Aarhus, Denmark; 6 Department of Global Public Health and Primary Care, University of Bergen, Bergen, Norway; 7 Division on Substance Use Disorders, New York State Psychiatric Institute, New York, New York, USA; 8 Department of Psychiatry, Columbia University Irving Medical Center, New York, New York, USA; 9 Department of Psychological and Brain Sciences, Indiana University Bloomington, Bloomington, Indiana, USA

**Keywords:** Attention Deficit and Disruptive Behavior Disorders, Substance-Related Disorders

## Abstract

**Background:**

Attention-deficit/hyperactivity disorder (ADHD) and substance use disorder (SUD) often coexist. ADHD complicates the course of disease in SUD. While recommended in guidelines, ADHD medication for those with comorbid SUD remains controversial.

**Objective:**

This study aims to explore how comorbid SUD affects ADHD medication discontinuation and reinitiation in individuals with ADHD.

**Methods:**

Using a matched cohort design, we identified 9283 individuals with ADHD and SUD in Sweden between 2006 and 2020, who had ongoing ADHD medication at the time of their first SUD-related event. Controls with ADHD medication but no SUD (n=46 401) were matched 5:1 on sex and birth year. HRs for treatment discontinuation within 1 year from first SUD, and for treatment reinitiation within 1 year from the first discontinuation, were estimated using stratified Cox models.

**Findings:**

Individuals with ADHD and SUD were nearly two times as likely to discontinue ADHD treatment within 1 year (HR: 1.99, 95% CI 1.92 to 2.07) and less likely to re-initiate ADHD treatment (HR: 0.82, 95% CI 0.76 to 0.89) compared with controls. Several factors, including male sex, adolescent to young adult age and lower stimulant medication dose, were associated with increased risk for treatment discontinuation, but only adolescent to young adult age was significantly associated with treatment reinitiation in those with ADHD and SUD.

**Conclusions:**

The results suggest less treatment continuity and access for those with ADHD and comorbid SUD.

**Clinical implications:**

Treatment providers need to consider the specific needs of individuals with ADHD and comorbid SUD, especially in young males, to improve treatment outcomes for vulnerable groups.

**WHAT IS ALREADY KNOWN ON THIS TOPIC**

- Substance use disorder (SUD) is a common comorbidity in individuals with attention-deficit/hyperactivity disorder (ADHD).
- International consensus statements recommend pharmacological ADHD treatment in parallel with SUD treatment, but ADHD medication in those with SUD is still controversial.

**WHAT THIS STUDY ADDS**

- In a Swedish national register-based matched cohort study, we found that comorbid SUD nearly doubled the risk for medication discontinuation and decreased reinitiation of ADHD medication compared with controls with ADHD but no SUD.
- Young adult age, male sex and lower stimulant medication dose were associated with increased risk for treatment discontinuation in those with ADHD and SUD.

**HOW THIS STUDY MIGHT AFFECT RESEARCH, PRACTICE OR POLICY**

- Results highlight the need to improve medication continuity for individuals with ADHD and SUD to optimise clinical outcomes.

## Background

 Numerous studies have described the comorbidity between attention-deficit/hyperactivity disorder (ADHD) and substance use disorder (SUD),^1^,^3^ indicating higher rates for hospitalisation, delinquency, psychiatric comorbidities and premature death in people with both conditions.^4 5^ Untreated ADHD complicates the course of SUD, hindering remission.^3 6^ International consensus statements recommend pharmacological ADHD treatment in parallel with SUD treatment,^3 6^ but ADHD medication in those with SUD is still controversial.^6^

Only a handful of randomised controlled studies have explored ADHD treatment in those with comorbid SUD,^7^ with conflicting results. Stimulants, constituting the first-line treatment in ADHD,^3 6 8^ have repeatedly shown lower effect sizes, more side effects and poorer treatment adherence in people with ADHD and comorbid SUD compared with those with ADHD alone.^9 10^ A more recent meta-analysis indicates small but positive effects on ADHD in those with comorbid ADHD and current SUD.^7^ Some evidence supports higher stimulant medication doses for ADHD in patients with concomitant ongoing stimulant (cocaine, methamphetamine or amphetamine) use disorders.^11 12^ However, the abuse potential of stimulant medications is of concern when treating these patient groups.^11^,^13^ Given that the risk for non-medical use and misuse of stimulant medications is in general more pronounced in SUD populations,^13 14^ guidelines recommend ADHD medications with lower abuse potential, such as extended-release stimulants or non-stimulant medications, in ADHD with comorbid SUD.^3 6^

In general, long-term adherence to ADHD medication is poor, with <50% of young adults still in treatment within a year after treatment initiation.^15^ A study based on a Danish registry indicates that SUD both delayed ADHD treatment initiation and increased the risk for treatment discontinuation.^16^ How the emergence and exacerbation of SUD affects ADHD medication discontinuation is largely unknown.

Several studies have explored risk factors associated with ADHD treatment discontinuation^15 17 18^ and reinitiation.^19^ Based on this literature, people with ADHD and SUD may have several risk factors for treatment discontinuation. The age of onset for most SUDs in adolescence and young adulthood corresponds to a period associated with increased risk for treatment discontinuation and decreased support in medication management.^18^ People with ADHD and SUD may also more often perceive ADHD medication side effects and less treatment effects,^9 10^ both associated with an increased risk for medication discontinuation.^15 17^ The increased risk for misuse and diversion of stimulant medication, especially in young males with ADHD and SUD,^13^ may further contribute to clinical decisions of treatment discontinuation when SUD is discovered.

Here, we used linkage of Swedish national registers to explore the risk of treatment discontinuation and reinitiation in people with ongoing ADHD medication, with and without SUD in a national cohort. In addition, we evaluated both individual and clinical practice-related factors associated with treatment discontinuation and reinitiation, including medication type, dose and psychiatric comorbidities.

## Methods

### Data source

Data were obtained by linking Swedish registers using the unique Swedish personal number. International Classification of Diseases (ICD) diagnoses were sourced from the National Patient Register (NPR), containing all inpatient and outpatient specialist care in Sweden, and dispensed medications by Anatomical Therapeutic Chemical (ATC) classification, from the Prescribed Drug Register (PDR). Information on SUD-related crime convictions was obtained from the National Crime Register; SUD-related death from the Cause of Death Register; demographic and socioeconomic variables from the Swedish Total Population Register (TPR). Registers and variables used are provided in online supplemental eTable 1.

### Study population

**Figure 1:**
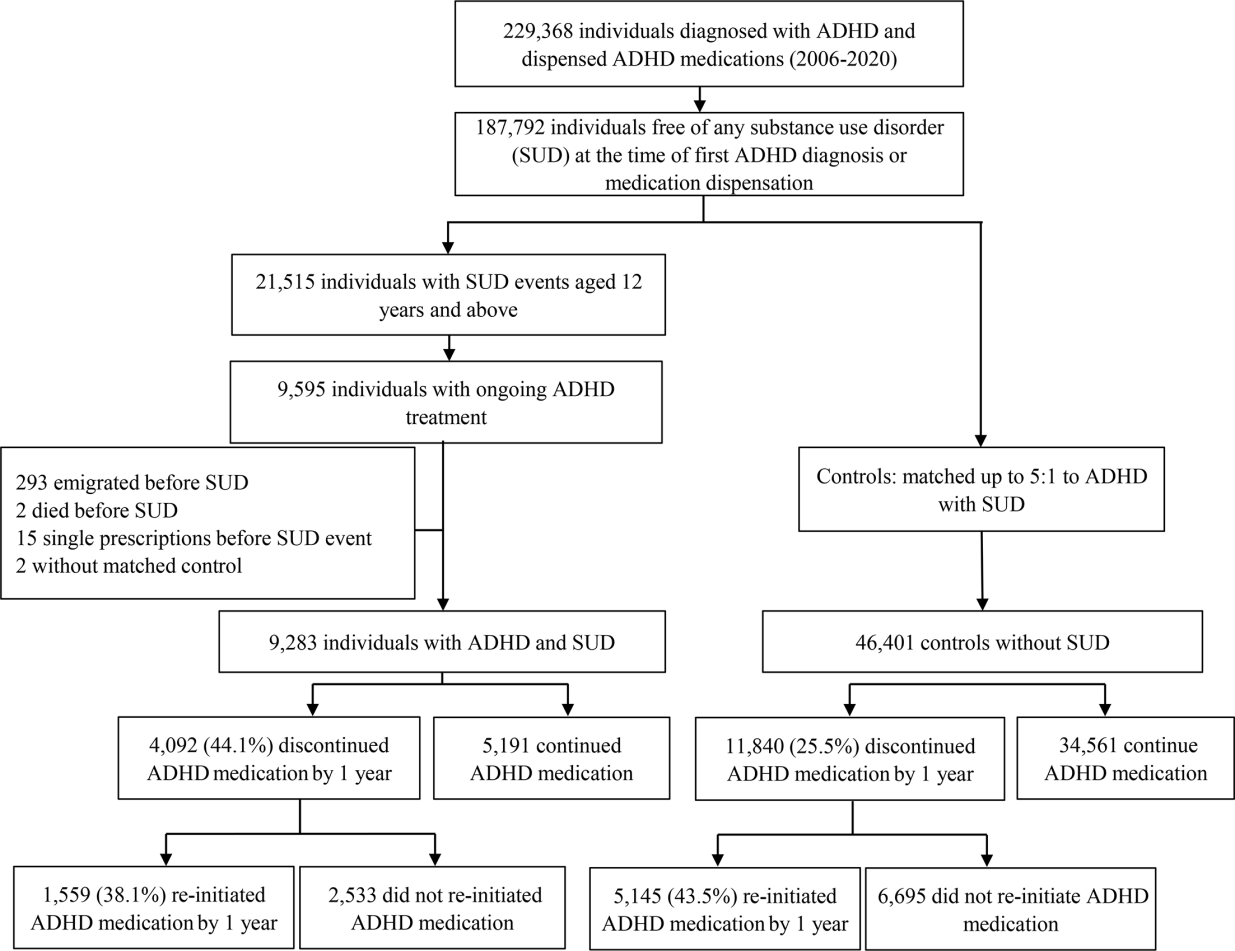
Overview of cohort inclusion for attention-deficit/hyperactivity disorder (ADHD) medication discontinuation and reinitiation.

From national registers, we first identified all individuals with an ADHD diagnosis who were dispensed stimulant (methylphenidate (MPH), amphetamine, dexamphetamine, lisdexamfetamine) and/or non-stimulant (atomoxetine and guanfacine) ADHD medication between 2006 and 2020 in Sweden (figure 1). Eligible individuals (n=187 792) had no documented SUD-related events (see below) at the time they received both their first ADHD diagnosis and their first medication dispensation. In Sweden, ADHD is diagnosed and medicated exclusively in specialised psychiatric or child and adolescent psychiatric care, rendering excellent coverage for ADHD diagnosis and medication in the registers.

*ADHD and SUD* were defined as having an ADHD diagnosis and ongoing medications at the time of their first SUD (online supplemental eFigure 1). For each individual with ADHD and SUD, up to five controls with ADHD diagnosis and medication but no SUD at the corresponding time were randomly selected and matched on sex, birth year and time for medication dispensation (calendar year and month) using incidence density sampling.^20^ As the objective of this study was to describe population-level prescription dispensing patterns rather than to draw causal inferences, we applied matching on key demographic and temporal factors combined with multivariable adjustment for relevant covariates. This approach ensured that controls were selected from the population at risk at the time each case occurred, preserving temporal comparability and minimising bias from time-dependent contextual factors such as changes in prescribing or diagnostic practices.^20^

### Measures

#### ADHD medication periods

ADHD medication periods were derived from a validated algorithm predicting treatment duration from free text in prescriptions.^21^ This method accounts for prescribed daily dosage and medication interruptions (such as weekends), enhancing accuracy compared with methods solely relying on medication dispensation dates. Medication doses were calculated using defined daily dose (DDD), defined as the assumed average maintenance dose per day for a drug used in adults for its main indication.^22^

#### ADHD medication discontinuation

ADHD medication discontinuation was defined as a treatment gap of ≥90 days without medication, calculated based on the estimated duration of the previous prescription (ie, days of supply). In general, in Sweden, prescriptions allow patients to pick up medication at pharmacies for a maximum of 3 months or less. Therefore, applying a 90-day treatment gap reduces the risk for misclassifying treated as untreated. Follow-up started on the date of the first SUD event for ADHD and SUD and the corresponding time-point for controls and ended at treatment discontinuation, 1 year from start of follow-up (2 years in the sensitivity analysis), end of study (31 December 2020), emigration or death, whichever came first. For controls, follow-up also ended on the date of any SUD event.

#### ADHD medication reinitiation

ADHD medication reinitiation was indicated by a subsequent ADHD medication dispensation following a treatment discontinuation. Follow-up for treatment reinitiation started at the date of discontinuation and ended at reinitiation, 1 year from discontinuation (2 years in sensitivity analysis), end of study, emigration, or death, or, for controls, any SUD event, whichever came first.

#### Substance use disorder (SUD)

Since various registers capture different aspects of SUD (social, medical, legal), using several registers renders a more accurate model of SUD in the population.^23^ SUD was therefore defined as the presence of one or more of the following: drug or alcohol related ICD diagnose code, medication for SUD, conviction for SUD-related criminal offence, or contact with specialised addiction care. This latter can be regarded as a proxy for SUD given that first-line treatment in primary healthcare is not included in the national registers and access to addiction care is low, mostly limited to the more severe or complex cases referred by primary healthcare and/or social services.^24^ SUD type was based on the specific ICD diagnosis code (online supplemental eTable 2). Stimulant use disorder was defined as either stimulant or cocaine use disorder, as amphetamine is the most common stimulant used in Sweden, while cocaine is less common compared with other European countries.^25^

#### Covariates

Covariates included baseline psychiatric, neurodevelopmental and medical comorbidities from the NPR (ie, anxiety, depressive, bipolar, psychotic, eating, personality, conduct or sleep disorders; autism, intellectual disability; cardiovascular disease, obesity, type 2 diabetes, dyslipidaemia; accidental injuries or poisoning) at the time of, or prior to the first SUD, or corresponding time for controls; the medical speciality of the treatment provider (psychiatry, child and adolescent psychiatry, addiction medicine, other) from the NPR; demographic factors (sex, birth year, country of birth, immigration, emigration) from the TPR, as well as any subsequent SUD event during follow-up.

### Statistical analysis

Stratified Cox regression models were used to estimate the HRs for ADHD medication discontinuation and reinitiation in those with ADHD and SUD compared with controls. The matched cohort design accounted for sex, birth year, time since last ADHD medication dispensation (calendar year and month). The models were further adjusted for age at ADHD diagnosis, country of birth, medical specialty of the prescriber and baseline psychiatric and medical comorbidities, as well as any subsequent SUD event during follow-up. Additionally, analyses were performed separately by sex.

In secondary analyses, within the ADHD and SUD cohort, we examined factors associated with treatment discontinuation and reinitiation, including sex, age at first SUD, ADHD medication dose expressed in DDDs at the index date, SUD type, psychiatric and medical comorbidities. We compared risk for discontinuation between different SUD diagnoses, using the group who had contact with addiction services as reference. Any additional SUD event during follow-up was included as a time-varying covariate. Additionally, among individuals re-initiating ADHD medication, we examined changes in ADHD medication type and prescribers’ medical specialty from discontinuation to reinitiation. We controlled for multiple testing using the false discovery rate method via the Benjamini-Hochberg procedure.^26^

Several sensitivity analyses were conducted to test the robustness of our findings. First, we probed if treated patients could be misclassified as untreated, as some medications in SUD services may be dispensed directly from the clinics, and therefore not recorded in the PDR. To this end, we examined the associations in the Stockholm region, where all outpatient medication has traditionally been prescribed to be collected at pharmacies. Second, we assessed changes in ADHD medication type after reinitiation after 2014, when lisdexamfetamine and guanfacine became available in Sweden. Third, we evaluated treatment discontinuation and reinitiation using a more restrictive SUD definition based solely on ICD diagnoses. Fourth, we explored alternative definitions for treatment discontinuation by applying 30-day and 60-day treatment gaps. Finally, we extended the follow-up period from one to 2 years.

This study was reported in accordance with the Strengthening the Reporting of Observational Studies in Epidemiology guidelines.

## Results

From the ADHD cohort with ongoing medication, we identified 9595 individuals. After excluding those who had emigrated, had died or only had collected ADHD medication once, we included 9283 (median age 19.2 years, IQR: 16.8–26.2) with ADHD and SUD and 46 401 matched controls (figure 1). SUD was identified through diagnosis in 55% (n=5130), 5.8% with medication only, 28% with SUD-related criminality and 11.3% through contact with specialised addiction care. Sample characteristics are presented in table 1. Stimulants were the most prescribed ADHD medications. Median (IQR) follow-up time for ADHD and SUD was 325 (110–366) days and for controls 365 (209–366) days. More than half of both ADHD and SUD (5531, 59.6%) and controls (25 078, 54.0%) had at least one psychiatric comorbidity. Psychiatric comorbidities, except for autism and intellectual disability, were more common in ADHD and SUD compared with controls.

**Table 1:** Baseline characteristics of individuals with ADHD medication with and without substance use disorder (SUD), matched for birth year and sex

Characteristics	ADHD and SUD N=9283	ADHD, but no SUD N=46 401	P value^§^*
Birth year, median (IQR)	1994 (1988–1999)	1994 (1988–1999)	0.97
Age at first SUD event† (years), median (IQR)	19.2 (16.8–26.2)	19.3 (16.8–26.1)	0.07
12–17	3387 (36.5%)	17 274 (37.5%)	
18–24	3340 (36.0%)	16 196 (35.2%)	
25 and above	2556 (27.5%)	12 543 (27.3%)	
Sex			0.98
Male	5563 (59.9%)	27 813 (59.9%)	
Female	3720 (40.1%)	18 588 (40.1%)	
Age at ADHD diagnosis (years)	16.0 (13.0–22.4)	16.0 (13.0–22.4)	<0.01
0–11	1810 (19.5%)	10 063 (21.7%)	
12–17	4073 (43.9%)	17 342 (37.4%)	
18–24	1478 (15.9%)	8904 (19.2%)	
25 and above	1922 (20.7%)	10 092 (21.7%)	
Birth country			0.96
Sweden	8610 (92.8%)	43 252 (93.2%)	
Other	673 (7.2%)	3149 (6.8%)	
ADHD medication type			<0.01
(Dex)amphetamine‡	23 (0.2%)	185 (0.4%)	
Atomoxetine	918 (9.9%)	5193 (11.3%)	
Dexamphetamine	265 (2.9%)	899 (2.0%)	
Guanfacine	154 (1.7%)	683 (1.5%)	
Lisdexamfetamine	1844 (19.9%)	6667 (14.5%)	
Methylphenidate	6079 (65.5%)	32 386 (70.4%)	
ADHD medication dose (mg)			
Amphetamine	30.0 (20.0–60.0)	30.0 (15.0–45.0)	0.31
Atomoxetine	50.0 (30.0–80.0)	60.0 (40.0–80.0)	0.02
Dexamphetamine	20.0 (10.0–35.0)	20.0 (10.0–40.0)	0.08
Guanfacine	2.0 (1.0–4.0)	2.0 (1.0–4.0)	0.22
Lisdexamfetamine	50.0 (30.0–60.0)	50.0 (30.0–60.0)	<0.01
Methylphenidate	36.0 (20.0–54.0)	36.0 (20.0–54.0)	<0.01
Defined daily dose (DDD)*	0.9 (0.5–1.3)	0.9 (0.5–1.3)	
Amphetamine	1.4 (1.0–1.7)	1.4 (0.7–2.4)	0.73
Atomoxetine	0.5 (0.4–0.7)	0.5 (0.3–0.7)	0.97
(Dex)amphetamine	1.0 (0.5–1.8)	1.0 (0.5–1.6)	0.74
Guanfacine	0.6 (0.4–1.0)	0.7 (0.4–1.0)	0.18
Lisdexamfetamine	1.2 (0.7–1.4)	1.2 (0.7–1.4)	0.58
Methylphenidate	0.9 (0.5–1.3)	0.9 (0.5–1.3)	0.08
SUD-related diagnoses and events			
Alcohol	2577 (27.8%)	–	
Cannabis	693 (7.5%)	–	
Opioids	194 (2.1%)	–	
Stimulants	256 (2.8%)	–	
Sedatives/hypnotics	483 (5.2%)	–	
Other	73 (0.7%)	–	
Multiple substance use	854 (9.2%)	–	
Substance-related crime	2573 (27.7%)	–	
SUD medication	535 (5.8%)		
Contact with specialised addiction care	1045 (11.3%)	–	
Baseline comorbid diagnoses			
Anxiety disorder	1297 (14.0%)	4909 (10.6%)	<0.01
Autism spectrum disorders	1461 (15.7%)	10 073 (21.7%)	<0.01
Bipolar disorder	660 (7.1%)	2283 (4.9%)	<0.01
Conduct disorder	864 (9.3%)	2438 (5.3%)	<0.01
Depressive disorder	3282 (35.4%)	12 000 (25.9%)	<0.01
Anorexia	109 (1.2%)	340 (0.7%)	<0.01
Bulimia	81 (0.9%)	239 (0.5%)	<0.01
Intellectual disability	433 (4.7%)	3884 (8.4%)	<0.01
Personality disorder	703 (7.6%)	2164 (4.7%)	<0.01
Schizophrenia	232 (2.5%)	616 (1.3%)	<0.01
Sleep disorders	772 (8.3%)	2953 (6.4%)	<0.01
Accidental poisoning	1505 (16.2%)	2752 (5.9%)	<0.01
Accidental Injuries	7137 (76.9%)	29 457 (63.5%)	<0.01
Cardiovascular diseases	443 (4.8%)	1883 (4.1%)	<0.01
Obesity	693 (7.5%)	3669 (7.9%)	0.14
Type 2 diabetes	78 (0.8%)	379 (0.8%)	0.81
Dyslipidaemia	40 (0.4%)	212 (0.5%)	0.73

Data are presented as median (IQR) or n (%).

SUD, defined as diagnosis (using ICD-9 and ICD-10 codes for specific SUD types and multiple substance use, multiple SUD denoted several ICD SUD diagnoses), medication (identified by Anatomical Therapeutic Chemical codes) or substance related criminal convictions; or contact with specialized addiction clinics: which denotes contact with specialized addiction services, but no ICD addiction diagnosis or medication.

Stimulant use disorders were combined to include both cocaine and other stimulant use disorders.

* DDD is based on the WHO values and corresponds to: 15 mg amfetamine; 15 mg dexamfetamine; 30 mg methylphenidate; 80 mg atomoxetine; 30 mg lisdexamfetamine; and 3 mg guanfacine.

† Age at first SUD or corresponding time for controls.

‡ (Dex)amphetamine denotes both dexamphetamine and amphetamine, the latter not approved for treatment in Sweden and only available on a special license from the medical products agency for certain individuals or treatment providers.

§ P values were calculated using t-tests for continuous variables and χ2 tests for categorical variables.

ADHD, attention-deficit/hyperactivity disorder; ICD, International Classification of Diseases; NA, not applicable.

### From SUD event to discontinuation of ADHD medication

Within 1 year from the first SUD or the corresponding time in controls, 44% of those with ADHD and SUD had discontinued treatment, compared with only 25% in the control group (online supplemental eFigure 2). Participants with SUD discontinued treatment on average 1 month earlier, with a median time to event of 111 (48–208) days compared with 142 days (71–239) in controls.

**Figure 2:**
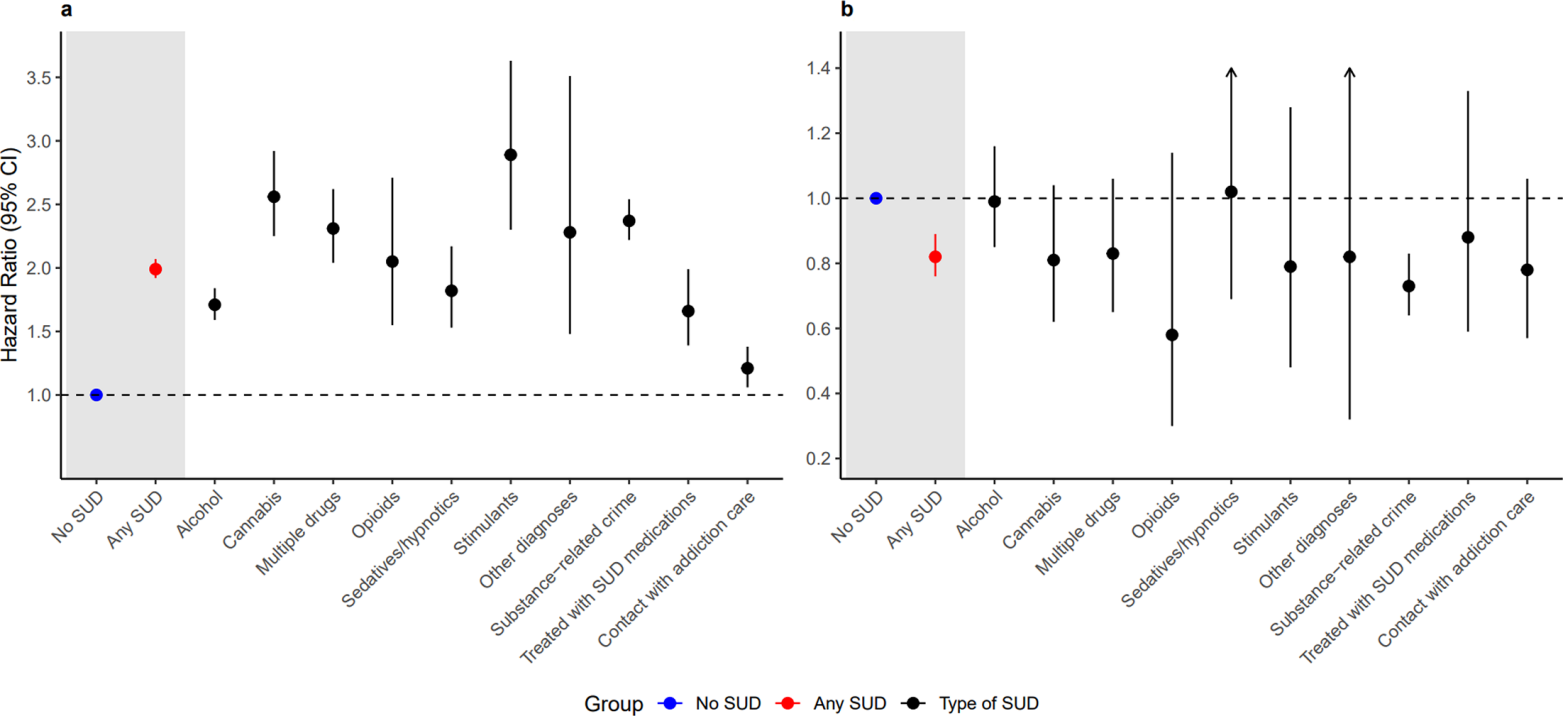
Association between substance use disorder (SUD) and attention-deficit/hyperactivity disorder (ADHD) medication discontinuation (a) and reinitiation (b). All models were adjusted for birth country, age at ADHD diagnosis, prescriber type and psychiatric and medical comorbidities at baseline and accounted for sex, birth year, time since last dispensation, calendar year and month.

Individuals with ADHD and SUD had a nearly twofold risk for treatment discontinuation within 1 year, compared with matched controls (adjusted HR: 1.99, 95% CI 1.92 to 2.07) (figure 2a). Males had significantly higher risks for treatment discontinuation compared with females (HR males: 2.17, 95% CI 2.06 to 2.28, HR females: 1.73, 95% CI 1.62 to 1.85), even after controlling for multiple comparisons.

Highest risk for treatment discontinuation was found in those with stimulant, cannabis and multiple drug use disorder, as well as in those with substance-related criminality (figure 2a and online supplemental eTable 3). Any subsequent SUD-related event further increased the risk for treatment discontinuation by 15% (1.15, 1.04 to 1.28) (table 2).

**Table 2:** Factors associated with ADHD medication treatment discontinuation and reinitiation among individuals with ADHD and comorbid SUD

Characteristics	Discontinuation	Reinitiation
	HR (95% CI)	HR (95% CI)
Age at ADHD diagnosis		
0–11	Reference	Reference
12–17	1.25 (1.15 to 1.36)*	0.86 (0.75 to 0.99)
18–24	1.17 (1.03 to 1.33)	0.85 (0.69 to 1.04)
25 and above	1.10 (0.91 to 1.33)	0.78 (0.57 to 1.05)
Age at first SUD event		
12–17	Reference	Reference
18–24	1.31 (1.18 to 1.46)*	0.70 (0.58 to 0.84)*
25 and above	1.03 (0.86 to 1.24)	0.81 (0.60 to 1.09)
Sex		
Male	Reference	Reference
Female	0.84 (0.79 to 0.91)*	1.17 (1.05 to 1.31)
Birth country		
Sweden	Reference	Reference
Other	1.10 (0.98 to 1.24)	0.96 (0.79 to 1.16)
Prescriber type		
Non-psychiatric specialist care	Reference	Reference
Adult psychiatry	0.81 (0.71 to 0.93)*	1.25 (1.00 to 1.55)
Child and adolescent psychiatry	1.03 (0.92 to 1.14)	0.92 (0.78 to 1.10)
Specialised SUD care	0.66 (0.43 to 1.00)	1.08 (0.56 to 2.08)
Criminal justice system	0.74 (0.56 to 0.99)	1.40 (0.88 to 2.22)
Primary healthcare	1.00 (0.79 to 1.26)	0.91 (0.61 to 1.35)
Defined daily doses (DDD)		
<0.5	Reference	Reference
0.5≤ and <1	0.94 (0.87 to 1.02)	1.03 (0.91 to 1.17)
1≤DDD<1.5	0.84 (0.77 to 0.91)*	0.98 (0.85 to 1.13)
≥1.5	0.66 (0.59 to 0.74)*	0.92 (0.76 to 1.10)
SUD type		
Contact with addiction care	Reference	Reference
Alcohol	1.43 (1.24 to 1.64)*	1.00 (0.81 to 1.25)
Cannabis	2.07 (1.76 to 2.44)*	0.89 (0.69 to 1.16)
Multiple drugs	1.90 (1.62 to 2.22)*	0.90 (0.70 to 1.15)
Opioids	1.63 (1.25 to 2.11)*	0.86 (0.57 to 1.31)
Sedatives/hypnotics	1.47 (1.21 to 1.78)*	0.90 (0.66 to 1.23)
Stimulants	2.18 (1.76 to 2.69)*	0.98 (0.70 to 1.38)
Other diagnoses	1.76 (1.22 to 2.53)*	1.08 (0.59 to 1.97)
Treated with SUD medications	1.17 (0.96 to 1.42)	1.03 (0.76 to 1.39)
Substance-related crime	2.07 (1.81 to 2.37)*	0.80 (0.64 to 1.00)
Any SUD event during follow-up	1.15 (1.04 to 1.28)	0.93 (0.78 to 1.11)
Baseline comorbid conditions		
Anxiety disorder	1.09 (0.99 to 1.21)	1.02 (0.87 to 1.19)
Autism spectrum disorder	0.86 (0.78 to 0.94)*	1.02 (0.88 to 1.18)
Bipolar disorder	1.00 (0.87 to 1.14)	1.06 (0.86 to 1.29)
Conduct disorder	1.04 (0.94 to 1.15)	0.88 (0.74 to 1.04)
Depressive disorder	0.91 (0.84 to 0.98)	0.97 (0.86 to 1.09)
Anorexia	0.93 (0.68 to 1.27)	1.11 (0.70 to 1.77)
Bulimia	0.93 (0.62 to 1.39)	0.80 (0.42 to 1.50)
Intellectual disability	0.87 (0.75 to 1.01)	0.67 (0.51 to 0.89)
Personality disorder	1.01 (0.88 to 1.16)	1.03 (0.84 to 1.26)
Schizophrenia	1.27 (1.05 to 1.55)	0.85 (0.62 to 1.17)
Sleep disorders	1.02 (0.91 to 1.14)	1.03 (0.86 to 1.24)
Accidental poisoning	1.03 (0.95 to 1.11)	0.94 (0.83 to 1.07)
Accidental Injuries	1.01 (0.92 to 1.11)	1.08 (0.93 to 1.24)
Cardiovascular diseases	0.89 (0.75 to 1.05)	0.97 (0.74 to 1.29)
Obesity	0.89 (0.78 to 1.01)	1.02 (0.82 to 1.26)
Type 2 diabetes	1.04 (0.70 to 1.53)	0.91 (0.46 to 1.78)
Dyslipidaemia	0.60 (0.31 to 1.16)	0.23 (0.03 to 1.64)

HRs and 95% CI were obtained from conditional Cox models adjusted for sex and any SUD event during follow-up included as a time-varying covariate using.

* Statistically significant after false discovery rate correction.

ADHD, attention-deficit/hyperactivity disorder; SUD, substance use disorder.

### From treatment discontinuation to reinitiation

A considerable proportion of both those with and without SUD re-initiated ADHD treatment within 1 year from discontinuation (figure 1). However, people with ADHD and SUD were significantly less likely to re-initiate ADHD treatment within 1 year compared with controls (HR: 0.82, 0.76–0.89) (figure 2b). Specifically, individuals with SUD-related criminality more seldom re-initiated ADHD treatment (HR: 0.73, 0.64–0.83) compared with controls (online supplemental eTable 4).

Compared with controls with ADHD but no SUD, people with ADHD and comorbid SUD were significantly more likely to change their medication type—particularly switching from MPH to other ADHD medications (p<0.01) (online supplemental eFigure 3) and specialty of treatment provider (online supplemental eFigure 4) after reinitiation, suggestive of lower continuity of care in these patients.

### Factors associated with treatment discontinuation and reinitiation in individuals with ADHD and SUD

Young adult age (18–24 years) at first SUD was significantly associated with higher treatment discontinuation and lower reinitiation (table 2).

Medication doses were inversely associated with treatment discontinuation. Higher doses (≥1 DDD) were associated with lower discontinuation risk, compared with low doses (<0.5 DDD). No statistically significant association was present with reinitiation (table 2). Both stimulant and non-stimulant medication was associated with increased risk for treatment discontinuation in individuals with ADHD and SUD (online supplemental eTable 5).

All SUD diagnoses significantly increased the risk for treatment discontinuation but not for treatment reinitiation (table 2). Autism was the only baseline comorbidity significantly associated with decreased risk for treatment discontinuation after controlling for multiple comparisons. None of the other comorbidities were significantly linked with treatment discontinuation or reinitiation.

### Sensitivity analyses

Medication discontinuation and reinitiation in the Stockholm region, where we expected a lower risk for misclassification of treated as untreated, showed similar results as in the main analysis (online supplemental eTable 5), similarly for the analyses with SUD defined strictly as a clinical diagnosis (online supplemental eFigure 5). When applying shorter treatment gaps (30 and 60 days) to define discontinuation, discontinuation rates increased among individuals both with and without SUD, whereas SUD remained associated with a higher likelihood of discontinuation and a lower likelihood of reinitiation. Finally, after 2-year follow-up, 58% of those with SUD discontinued ADHD treatment compared with only 37% of controls (online supplemental eTable 6).

## Discussion

We explored ADHD medication discontinuation and reinitiation in a national ADHD cohort, comparing individuals with comorbid SUD to matched controls without SUD. We found a nearly twofold risk for treatment discontinuation in ADHD and SUD compared with controls. All different SUD types were associated with treatment discontinuation, as was subsequent SUD-related event. Although a significant proportion of individuals, both with and without SUD, re-initiated ADHD treatment within 1 year, those with SUD re-initiated to a lesser extent. Several individual factors such as male sex, young adult age and stimulant medication dose showed statistically significant association with treatment discontinuation, and young adult age with lower treatment reinitiation in those with ADHD and SUD.

Young adult age has been described in earlier research regarding ADHD as a significant risk for medication discontinuation.^15^ In line with earlier publications, both psychiatric and medical comorbidities, as well as accidental poisonings, showed higher prevalence in those with ADHD and SUD compared with controls.^4 5^ Psychiatric and cardiovascular comorbidities, common in those with ADHD and SUD, may increase the risk for adverse medication effects,^4 9 10^ and patients and clinicians alike may choose to discontinue treatments perceived as potentially harmful. Contrary to our hypothesis, there was no significant association between psychiatric or medical comorbidities and treatment discontinuation and reinitiation in those with ADHD and SUD, with the notable exception of autism significantly associated with decreased treatment discontinuation risk.

We also found that somewhat higher stimulant medication doses at levels above 1 DDD were significantly associated with lowered risk for treatment discontinuation in ADHD and SUD. This could in part reflect patient selection, as patients who tolerate stimulants well, including in higher doses, are more likely to continue to remain on treatment. The findings are also in line with earlier research suggesting that somewhat higher stimulant medication doses may be necessary for treating patients with comorbid SUD.^11 12^

Individuals with ADHD and SUD more often changed treatment provider between discontinuation and reinitiation, compared with controls, suggestive of lower continuity of care. SUD patients, especially those with more severe SUD, have difficulties accessing and engaging with the healthcare system, due to stigma and low health literacy.^27^ Such factors, although impossible to explore using register data, may contribute to the increased risk of treatment discontinuation and lower reinitiation.

People with SUD-related criminal convictions constitute a particularly vulnerable group regarding treatment discontinuation. ADHD patients, especially with early onset SUD and a comorbid conduct disorder, are at a higher risk for criminality.^5^ Research indicates a lower risk for criminality in periods with ADHD treatment compared with those without.^28^ Thus, treating ADHD in those with criminal convictions may be particularly important. We found a significantly higher risk for treatment discontinuation in people with SUD-related criminality compared with controls. These results are in line with reviews indicating that ADHD is often overlooked in prison populations and seldom treated appropriately.^5^ Whether this is due to patients experiencing difficulties engaging and adhering to treatment regimens or whether healthcare providers are reluctant to prescribe stimulants to patients with ADHD and SUD,^29^ in the presence of criminal history, or consider treating this patient group riskier and therefore more often discontinue treatment is unclear. It is, however, increasingly clear that healthcare services need to be adapted to the specific needs of patients with ADHD, SUD and criminality to reach more patients and improve long-term outcomes.

### Strengths and limitations

A strength of this work is that it is based on a national sample of real-life data with very little or no attrition. This allows for a long-term follow-up of the whole spectrum of ADHD and SUD, including those with comorbidities who would be too unstable for or excluded from participating in clinical trials or other observational studies.

There are, however, several limitations. First, defining treatment discontinuation and reinitiation based on register data is inherently challenging,^30^ given that we do not know to what extent dispensed medications were in fact taken. In other words, medication as described in a pharmacoepidemiologic study is not a measure of medication intake or adherence. Data on medication are based on dispensed prescriptions that serve as a proxy for ongoing medication treatment rather than a direct measure of treatment adherence or medication use. We used a conservative definition of treatment discontinuation, allowing a long gap between dispensations to better capture true treatment interruptions. However, it is still possible for discontinuation to be misclassified, especially in younger children, where ADHD medication is more often prescribed with certain breaks for weekends or school holidays and among participants who take their medication more infrequently. Second, the registers most often do not contain information on reasons for treatment discontinuation. In some cases, treatment may be discontinued due to remission of ADHD. Discontinuation may also reflect the result of medical decisions due to lack of effects or increased risk for side effects. Third, patients with SUD may be dispensed medication from the addiction clinics, and therefore some participants classified as untreated, that is, discontinued, may in fact continue receiving supervised medication from a clinic. However, comparing a region, where all medications, including medications for SUD, are dispensed through pharmacies, with the rest of the country showed no significant difference in treatment discontinuation or reinitiation. Fourth, register-based data tends to capture the more severe end of the SUD spectrum. While all ADHD is diagnosed and treated in specialised care in Sweden and thus included in our data, harmful alcohol use and milder forms of alcohol use disorder are treated in primary care and therefore not captured in national registers. This may limit generalisability to the less severe, non-treatment seeking SUD cases, as well as to those with SUD onset before ADHD is diagnosed. Also, those who discontinued ADHD medication before an SUD event occurred or was registered were not included. This would mean that the number of discontinuations we capture is an underestimation and that our estimates are likely to be conservative. It would, however, not change the direction of the association. And finally, the findings may not be generalisable to other settings due to differences in healthcare access, diagnostic criteria and prescribing practices across populations.

In summary, although international consensus statements recommend treatment for ADHD in those with comorbid SUD, our results show that this population is at an increased risk of discontinuing ADHD medication and less likely to re-initiate treatment. Given that treatment adherence has been associated with positive outcomes in both ADHD and SUD, it is important to improve treatment access and continuity of care, especially in the susceptible period during the transition between adolescence and adulthood, a period marked by heightened vulnerability for both SUD onset and for treatment discontinuation.

## Supplementary material

10.1136/bmjment-2025-302138 online supplemental file 1

## Data Availability

Data may be obtained from a third party and are not publicly available.
