# Supplementary material for: Discontinuation and reinitiation of pharmacological treatment for ADHD among individuals with ADHD and substance use disorder
Source: BMJ Ment Health. 2026 Mar 23;29(1):e302138. doi: 10.1136/bmjment-2025-302138 (PMC13034277; doi:10.1136/bmjment-2025-302138)
Supplement: online supplemental file 1 [file bmjment-29-1-s001.docx]

**Supplemental materials**

**eTable 1** Overview of variables from the different Swedish national registers used in the project.

| Register (year coverage) | Type of data | Variables extracted |
| --- | --- | --- |
| National Patient Register (NPR) includes inpatients since 1973- and outpatients since 2001- ^1^ | In- and outpatient physician visits in specialized health care services, including, dates for the visits, types of care, diagnoses for psychiatric and medical disorders coded according to the International Classification of Disease (ICD) in eighth (ICD-8; 1969-1986), ninth (ICD-9; 1987-1996), and tenth (ICD-10; since 1997) revisions | SUD: all ICD 10 F1 except F17(nicotine);  ADHD: ICD -10: F90, ICD-9: 314;  Comorbid conditions: **Psychiatric comorbidities:**  Schizophrenia F20-F29; Bipolar disorder: F30-F31; Depression F32-F33; Anxiety disorders: 40-F41; Anorexia: F500, F501; Bulimia: F502, F503; Personality disorders F60, F69; Intellectual disability; F70-F79; Autism spectrum disorders: F84; Conduct disorder:F91; Sleep disorder F51, G47.0- G47.4, G47.8, G47.9  **Medical comorbidities:**  Cardiovascular disease: I00-I70, I73.0, I74-I75; Type 2 diabetes E11; Dyslipidemia: E78; obesity E66  **Accidental poisoning:** X40-49, T36-51; **Accidental Injuries:** S, T  **Contact with addiction services**  (codes for types of specialized services):  945 Alcohol abuse health care,  953 Addiction care,  954 Drug addiction care |
| Prescribed Drug Register (PDR) since 2005- | Information on dispensed drugs, registered according to the Anatomical Therapeutic Chemical (ATC) Classification codes | ADHD medication:  **Stimulants:**  methylphenidate [ATC: N06BA04], amphetamine [N06BA01], dexamphetamine [N06BA02], lisdexamphetamine [N06BA12])  **Non-stimulants:**  atomoxetine [N06BA09]  guanfacine [C02AC02]  **SUD medication:**  alcohol addiction [N07BB] including disulfiram, naltrexone, acamprosate and nalmefene,  opioid addiction [N07BC] including buprenorphine, buprenorphine/naloxone combinations, methadone, and levomethadone |
| National Cause of Death Register (since 1952- ) ^2^ | Death dates with underlying and contributory causes of death, registered by ICD codes | Death date, Cause of death, cause of death related to alcohol and or drugs |
| Longitudinal integration database for health insurance and labour market studies (LISA) since 1990-^3^ | Yearly information on education, employment, income, and social welfare for individuals ≥15 years old | Highest achieved education level |
| National Register of Crime Convictions, since 1973-^4^ | Information on crime convictions from Swedish general courts for individuals ≥15 years | Convictions for substance related offences |
| Total Population Register (TPR), since 1968 ^5^ | Covers demographic information on Swedish inhabitants since 1968, including migrations in/out of Sweden | Sex, age, country of birth, residence, and im-/emigration |

Registers in Sweden, a service provided by the Swedish Research Council <https://www.registerforskning.se/en/registers-in-sweden/> accessed 2025-01-08

**References:**

1. Ludvigsson JF, Andersson E, Ekbom A, et al. External review and validation of the Swedish national inpatient register. *BMC Public Health*. 2011;11(1):450.

2. Brooke HL, Talbäck M, Hörnblad J, et al. The Swedish cause of death register. *European journal of epidemiology*. Sep 2017;32(9):765-773. doi:10.1007/s10654-017-0316-1

3. Ludvigsson JF, Svedberg P, Olén O, Bruze G, Neovius M. The longitudinal integrated database for health insurance and labour market studies (LISA) and its use in medical research. *Eur J Epidemiol*. Apr 2019;34(4):423-437. doi:10.1007/s10654-019-00511-8

4. National Council for Crime Prevention. Kriminalstatistik 2010 [Criminal

statistics 2010]. *National Council for Crime Prevention*. 2010;

5. Ludvigsson JF, Almqvist C, Bonamy A-KE, et al. Registers of the Swedish total population and their use in medical research. *European Journal of Epidemiology*. 2016;31(2):125-136.

## **eTable 2**. International Classification of Diseases (ICD) diagnose codes and type of treatment provider from the National Patient Register and Anatomical Therapeutic Chemical (ATC) codes form the Prescribed Drug Register for SUD, and Cause of Death Register.

|  | ICD-10 codes/ATC codes |
| --- | --- |
| **SUD diagnosis (ICD codes)** |  |
| Alcohol | F10 |
| Cannabis | F12 |
| Multiple drugs | F19 |
| Opioids | F11 |
| Sedatives/hypnotics | F13 |
| Stimulants | F14-15 |
| Other diagnoses | F16, F18 |
| **SUD medication (ATC codes)** |  |
| Alcohol | N07BB |
| Opioid use disorder | N07BC |
| **Contact with addiction care** |  |
| 945 | ALKOHOLSJUKVÅRD |
| 953 | TOXIKOMANIVÅRD |
| 954 | NARKOMANVÅRD |
| **SUD-related crime** | Substance related offenses |

**eTable 3.** Factors associated with **ADHD medication** **discontinuation** in a national ADHD cohort with medication for ADHD, with concomitant substance use disorder (SUD) and sex and age matched controls with ADHD medication but no SUD.

| Characteristics |  | HR (95% CI)^b^ |
| --- | --- | --- |
| SUD type | No SUD | 1.0 |
|  | **Alcohol** | **1.71 (1.59-1.84)*** |
|  | **Cannabis** | **2.56 (2.25-2.92)*** |
|  | **Multiple drugs** | **2.31 (2.04-2.62)*** |
|  | **Opioids** | **2.05 (1.55-2.71)*** |
|  | **Sedatives/hypnotics** | **1.82 (1.53-2.17)*** |
|  | **Stimulants** | **2.89 (2.30-3.63)*** |
|  | **Other diagnoses** | **2.28 (1.48-3.51)*** |
|  | **Substance-related criminal offence** | **2.37 (2.22-2.54)*** |
|  | **Treated with SUD medications** | **1.66 (1.39-1.99)*** |
|  | **Contact with addiction care** | **1.21 (1.06-1.38)** |
| Comorbidities^a^ |  |  |
|  | Anxiety disorder | 0.99 (0.93-1.06) |
|  | **Autism spectrum disorders** | **0.78 (0.75-0.82)*** |
|  | Bipolar disorder | 0.97 (0.88-1.06) |
|  | Conduct disorder | 1.02 (0.95-1.10) |
|  | Depressive disorder | 0.97 (0.93-1.01) |
|  | **Anorexia** | **0.72 (0.58-0.90)** |
|  | Bulimia | 0.85 (0.65-1.12) |
|  | **Intellectual disability** | **0.55 (0.51-0.60)*** |
|  | Personality disorder | 1.04 (0.95-1.15) |
|  | Schizophrenia | 1.15 (0.99-1.35) |
|  | Sleep disorders | 1.02 (0.94-1.10) |
|  | Accidental Injuries | 1.02 (0.98-1.06) |
|  | Accidental poisoning | 0.94 (0.87-1.01) |
|  | Cardiovascular diseases | 0.93 (0.84-1.03) |
|  | **Obesity** | **0.86 (0.80-0.93)*** |
|  | Type 2 diabetes | 1.00 (0.78-1.28) |
|  | Dyslipidemia | 0.92 (0.66-1.29) |

^a^ Baseline comorbidity, diagnoses assessed at first SUD event or corresponding time for controls;  ^b^HR (95% CI): hazard ratio (95% confidence interval).Models adjusted for birth country, age at ADHD diagnosis, medical speciality of the prescriber, and accounted for sex, birth year, time since last dispensation (calendar year and month) in addition to matching variables in the matched cohort design. Bold font indicates statistical significance and * statistical significance after correcting for multiple comparisons according to the Benjamini-Hochberg FDR method.

**eTable 4.** Association between individual SUD and ADHD medication reinitiaion among individuals with ADHD medication treatment.

| Characteristics |  | HR (95% CI)^b^ |
| --- | --- | --- |
| SUD type | No SUD | 1.0 |
|  | Alcohol | 0.99 (0.85-1.16) |
|  | Cannabis | 0.81 (0.62-1.04) |
|  | Multiple drugs | 0.83 (0.65-1.06) |
|  | Opioids | 0.58 (0.30-1.14) |
|  | Sedatives/hypnotics | 1.02 (0.69-1.50) |
|  | Stimulants, including cocaine | 0.79 (0.48-1.28) |
|  | Other diagnoses | 0.82 (0.32-2.13) |
|  | **Substance-related criminal offence** | **0.73 (0.64-0.83)*** |
|  | Treated with SUD medications | 0.88 (0.59-1.33) |
|  | Contact with addiction care | 0.78 (0.57-1.06) |
| Comorbidities^a^ |  |  |
|  | Anxiety disorder | 0.89 (0.78-1.01) |
|  | Bipolar disorder | 0.91 (0.74-1.11) |
|  | **Conduct disorder** | **0.74 (0.63-0.87)*** |
|  | Depressive disorder | 0.96 (0.87-1.05) |
|  | Anorexia | 1.09 (0.69-1.72) |
|  | Bulimia | 0.82 (0.47-1.43) |
|  | Personality disorder | 1.21 (0.99-1.49) |
|  | Schizophrenia | 0.81 (0.57-1.16) |
|  | **Sleep disorders** | **1.20 (1.03-1.40)** |
|  | Autism spectrum disorders | 0.93 (0.84-1.03) |
|  | Intellectual disability | 0.86 (0.72-1.04) |
|  | Accidental Injuries | 0.97 (0.90-1.05) |
|  | Accidental poisoning | 1.05 (0.91-1.22) |
|  | Cardiovascular diseases | 0.91 (0.72-1.15) |
|  | Obesity | 1.02 (0.87-1.18) |
|  | Type 2 diabetes | 0.82 (0.47-1.44) |
|  | Dyslipidemia | 0.53 (0.19-1.47) |

^a^ Baseline comorbidity, diagnoses assessed at first SUD event or corresponding time for controls;  ^b^HR (95% CI): hazard ratio (95% confidence interval).Models adjusted for birth country, age at ADHD diagnosis, medical speciality of the prescriber, and accounted for sex, birth year, time since last dispensation (calendar year and month) in addition to matching variables in the matched cohort design.

Bold font indicates statistical significance and * statistical significance after correcting for multiple comparisons according to the Benjamini-Hochberg FDR method.

**eTable 5**. Proportion of discontinuation and reinitiation in sensitivity analyses at 1 year follow up.

|  | Discontinuation (%) | Re-initiation (%) |
| --- | --- | --- |
| **Restrict to Stockholm region** |  |  |
| Individuals with ADHD and SUD | 1050 (48.0%) | 429 (40.9%) |
| Controls without SUD | 3167 (29.6%) | 1452 (45.8%) |
| **SUD defined by clinical diagnosis*** |  |  |
| Individuals with ADHD and SUD | 3091 (45.5%) | 1165 (37.7%) |
| Controls without SUD | 8588 (25.3%) | 3711 (43.2%) |
| **By medication type** |  |  |
| **Stimulants** |  |  |
| Individuals with ADHD and SUD | 3588 (43.7%) | 1404 (39.1%) |
| Controls without SUD | 10185 (25.2%) | 4607 (45.2%) |
| **Non-stimulants** |  |  |
| Individuals with ADHD and SUD | 504 (47.1%) | 155 (30.8%) |
| Controls without SUD | 1655 (28.3%) | 538 (32.5%) |

* Substance use disorder (SUD) was defined as any ICD-9 or ICD-10 alcohol- or substance-related diagnosis, excluding nicotine.

.**eTable 6**. Sensitivity analysis of the association between SUD and ADHD medication discontinuation and reinitiation with alternative definitions of discontinuation.

|  | Discontinuation (%) | HR (95% CI) | Re-initiation (%) | HR (95% CI) |
| --- | --- | --- | --- | --- |
| **Treatment discontinuation defined by a gap of 60 days** |  |  |  |  |
| Individuals with ADHD and SUD | 5315 (57.3%) | 1.74  (1.69-1.81) | 2141 (40.3%) | 0.79  (0.74-0.85) |
| Controls without SUD | 18442 (39.7%) | 1 | 7445 (40.4%) | 1 |
| **Treatment discontinuation defined by a gap of 30 days** |  |  |  |  |
| Individuals with ADHD and SUD | 5926 (63.8%) | 1.67  (1.61-1.72) | 3020 (51.0%) | 0.75  (0.71-0.79) |
| Controls without SUD | 21729 (46.8%) | 1 | 11601 (53.4%) | 1 |

**eTable 7**. Proportion of discontinuation and reinitation of ADHD medication among individuals with ADHD and comorbid SUD, by types of SUD, at 1 year and 2 years follow up.

| **Type of SUD** | **N** | Discontinuation at 1yr (%) | Re-initiation at 1 yr (%) | Discontinuation at 2 yrs (%) | Re-initiation at 2 yrs (%) |
| --- | --- | --- | --- | --- | --- |
| **Controls** |  |  |  |  |  |
| **Any SUD** | 9,283 | 4,092 (44.1%) | 1,559 (38.1%) | 5,384 (58.0%) | 2,630 (48.9%) |
| Alcohol | 2,577 | 1,023 (39.7%) | 439 (42.9%) | 1,405 (54.5%) | 759 (54.0%) |
| Cannabis | 693 | 379 (54.7%) | 142 (37.5%) | 490 (70.7%) | 228 (46.5%) |
| Sedatives/hypnotics | 483 | 183 (37.9%) | 72 (39.3%) | 256 (53.0%) | 124 (48.4%) |
| Other stimulants* | 231 | 115 (49.8%) | 45 (39.1%) | 136 (58.9%) | 69 (50.7%) |
| Opioids | 194 | 74 (38.1%) | 28 (37.8%) | 96 (49.5%) | 54 (56.3%) |
| Hallucinogens | 50 | 27 (54.0%) | 11 (40.7%) | 32 (64.0%) | 15 (46.9%) |
| Cocaine | 25 | 12 (48.0%) | 4 (33.3%) | 15 (60.0%) | 6 (40.0%) |
| Volatile solvents | 23 | 6 (26.1%) | 1 (16.7%) | 10 (43.5%) | 5 (50.0%) |
| Multiple drugs | 854 | 402 (47.1%) | 150 (37.3%) | 501 (58.7%) | 263 (52.5%) |
| Substance related crime | 2,573 | 1,401 (54.5%) | 472 (33.7%) | 1,788 (69.5%) | 774 (43.3%) |
| Treated with SUD medications | 535 | 170 (31.8%) | 75 (44.1%) | 241 (45.0%) | 126 (52.3%) |
| Contact with addiction care | 1,045 | 300 (28.7%) | 120 (40.0%) | 414 (39.6%) | 207 (50.0%) |

SUD: substance use disorder, including alcohol use disorder;

*Other stimulants: include amphetamine, methamphetamine, cathinones

# **eFigure 1:** Follow up of the study population

Outcome:

discontinuation

T0: Corresponding time

On treatment

(defined by without treatment gap of 90) days or more

T0: SUD

Last ADHD medication dispensation

Unexposed*

Exposed

Outcome:

reinitiation

*matched on sex, birth year, time since last medication dispensation (calendar year and month).

Eligible individuals without SUD had to be alive, living in Sweden, and free of SUD at the time when their matched individual with SUD had a SUD event.

# **eFigure 2.** Cumulative incidence of treatment discontinuation in individuals with ADHD and SUD compared to controls.


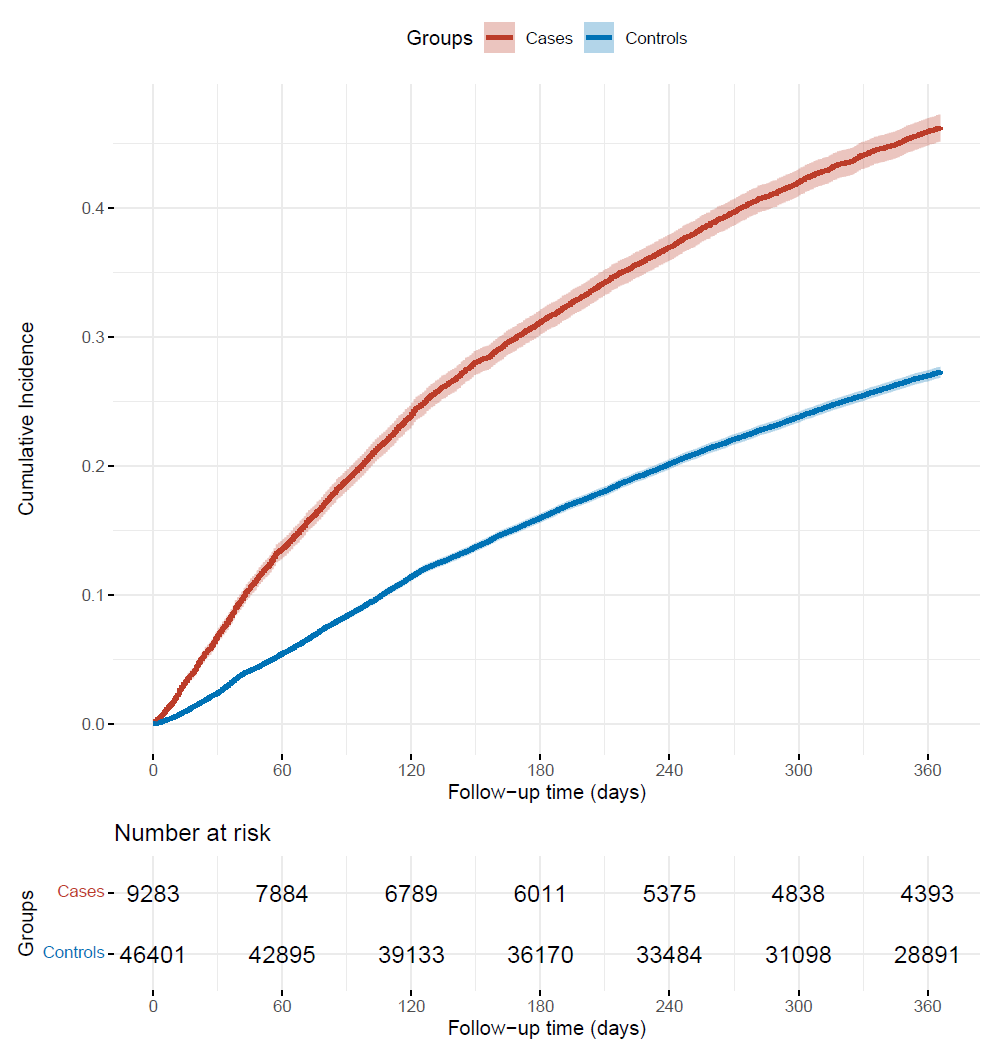


X axis represents time in days from first SUD related event or corresponding time in controls to discontinuation of ADHD medication.

**eFigure 3.** Sankey plot for change of ADHD medication type from discontinuation to re-initiation

Individuals diagnosed with ADHD and dispensed ADHD medications between 2006-2020

**ADHD and SUD (cases) ADHD without SUD (controls)**


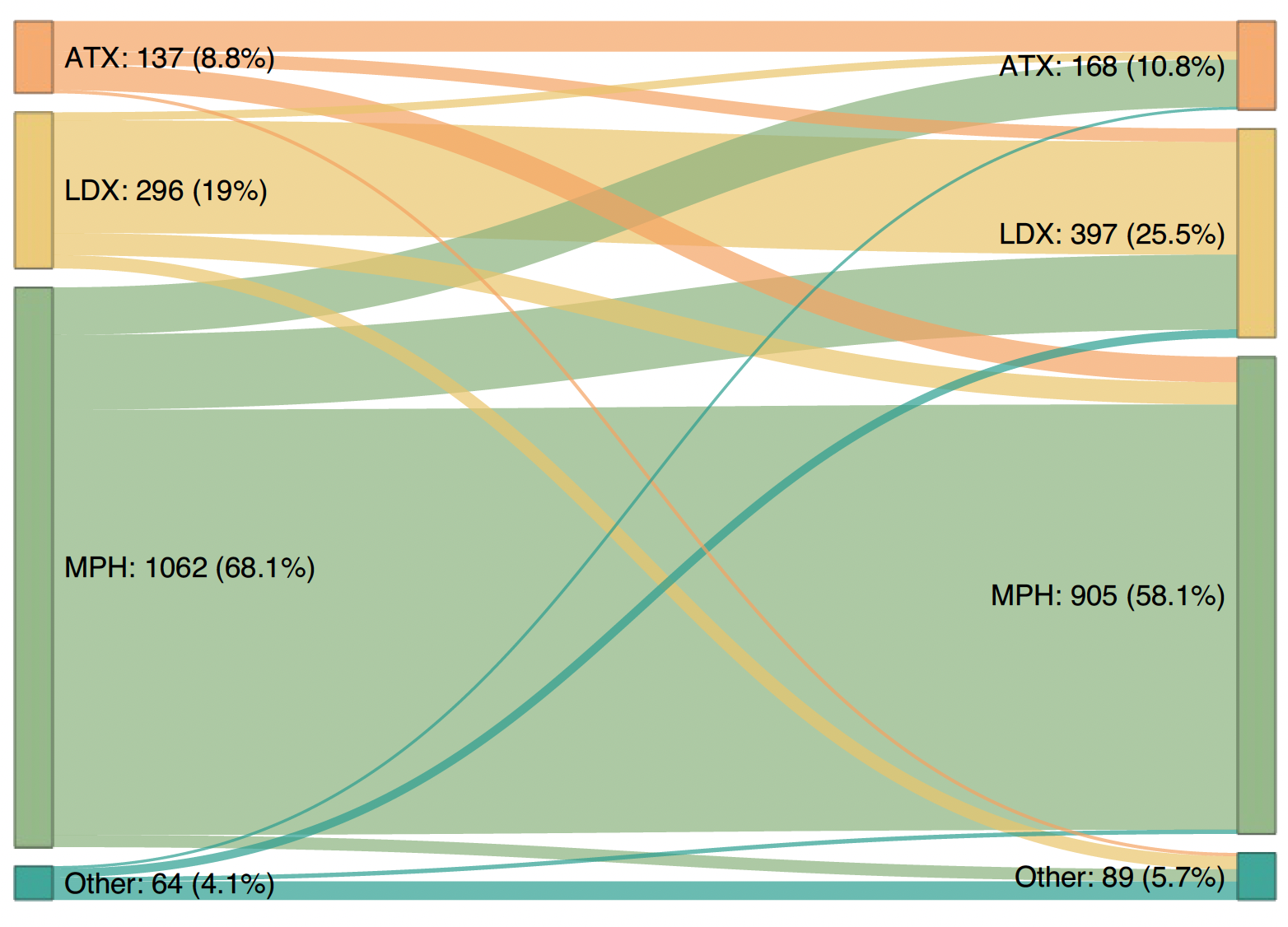

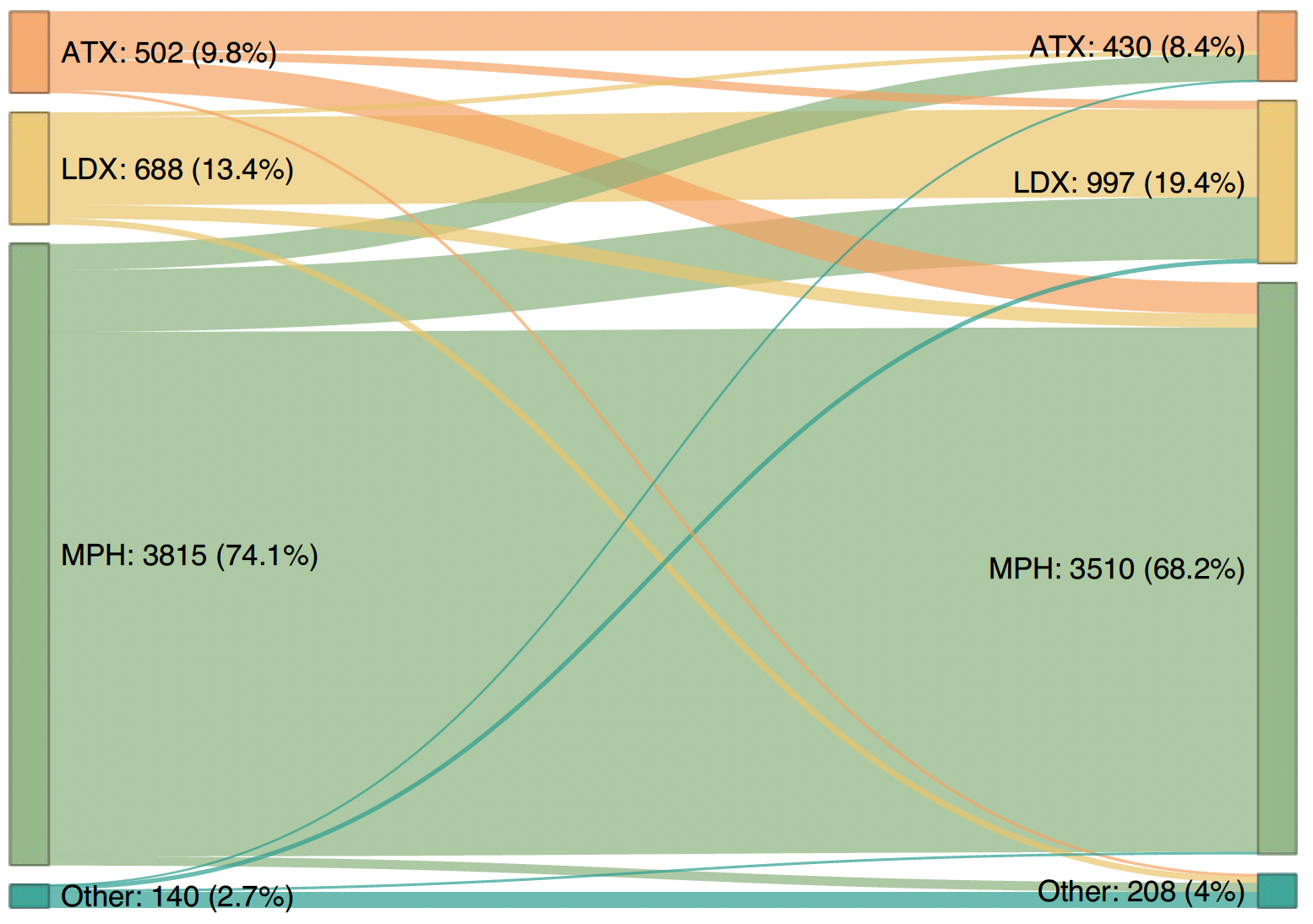


(Test ADHD and SUD vs controls in terms of proportion of change from MPH to other types of ADHD medication, p<0.01)

Individuals diagnosed with ADHD and dispensed ADHD medications between 2014-2020, when lisdexamphetamine and guanfacine were introduced in Sweden

**ADHD and SUD (cases) ADHD without SUD (controls)**

**
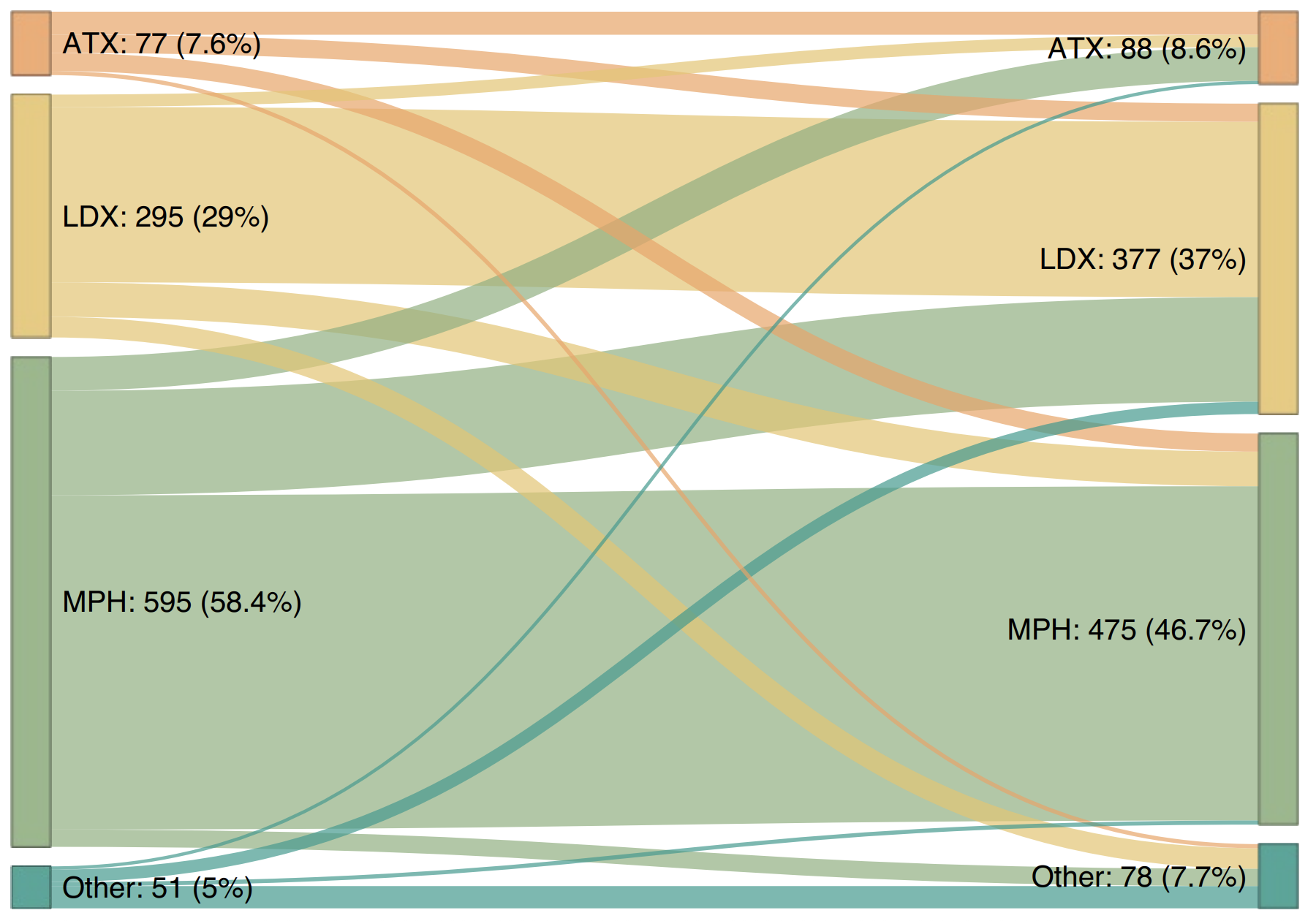

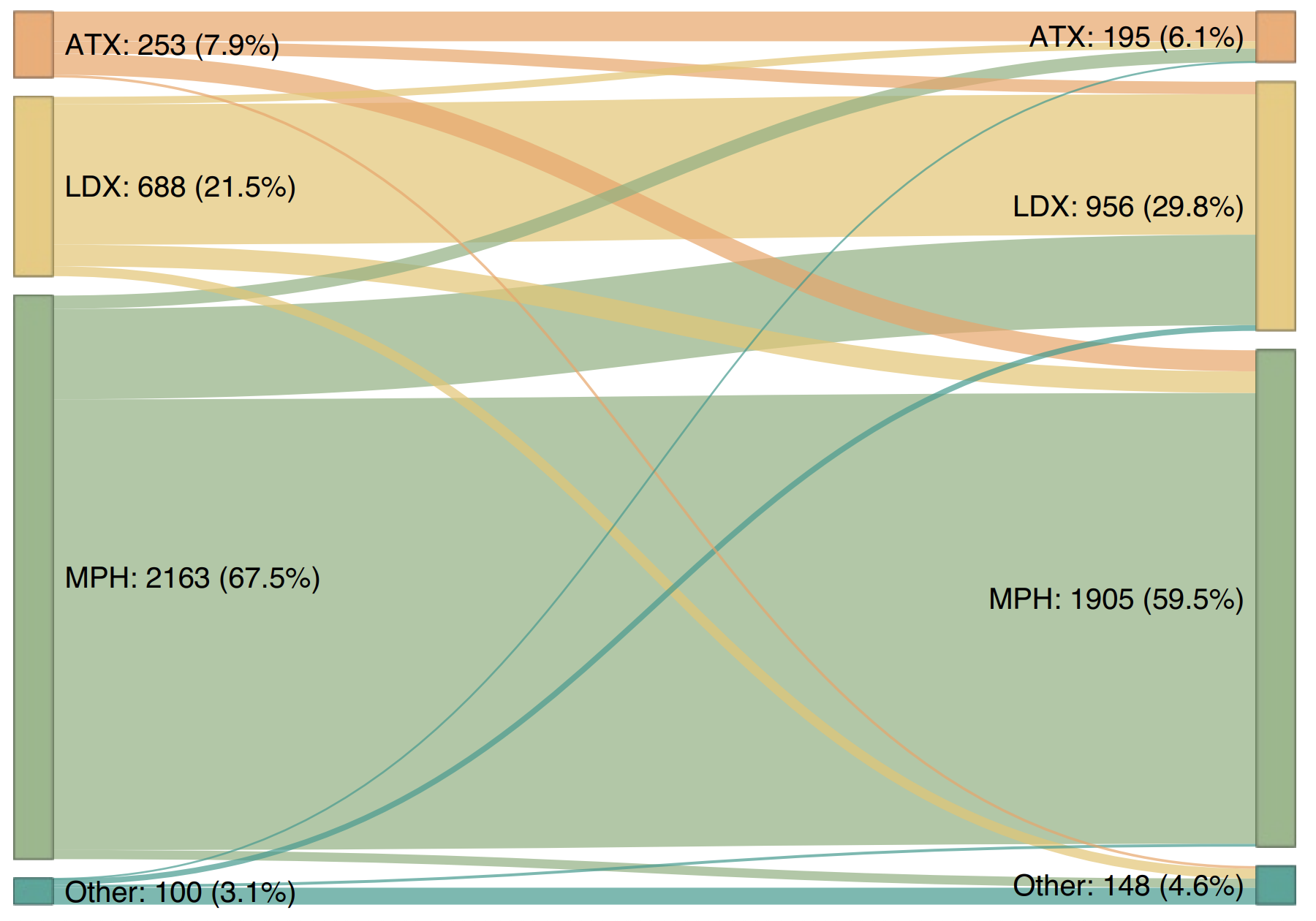
**

(Test ADHD and SUD vs controls in terms of proportion of change from MPH to other types of ADHD medication, p<0.01)

MPH: methylphenidate; LDX: lisdexamphetamine; ATX: atomoxetine; Other: guanfacine, dexamphetamine, amphetamine

**eFigure 4.** Sankey plot for change of medical specialty of treatment provider from discontinuation to re-initiation

**ADHD and SUD (cases) ADHD without SUD (controls)**
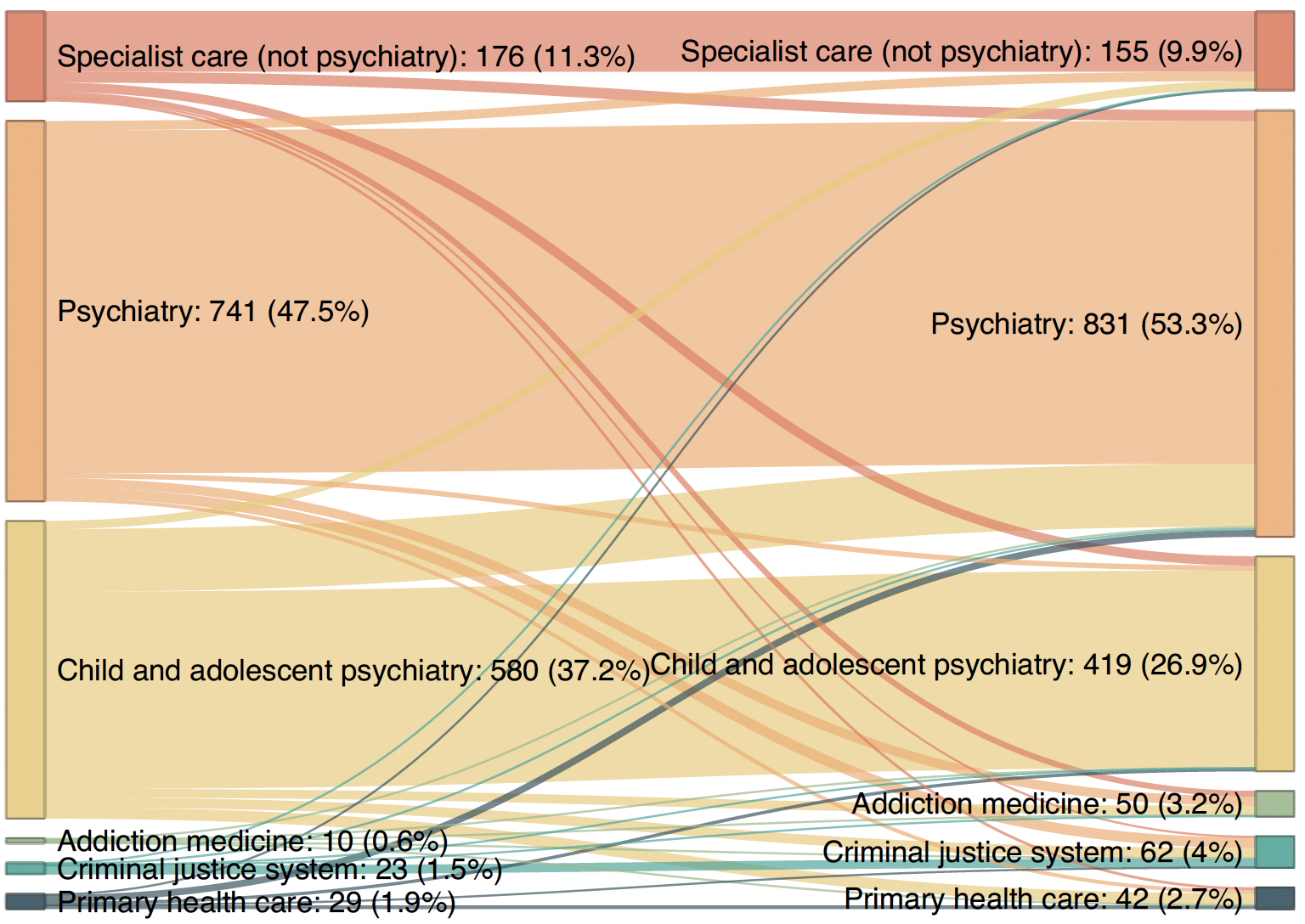

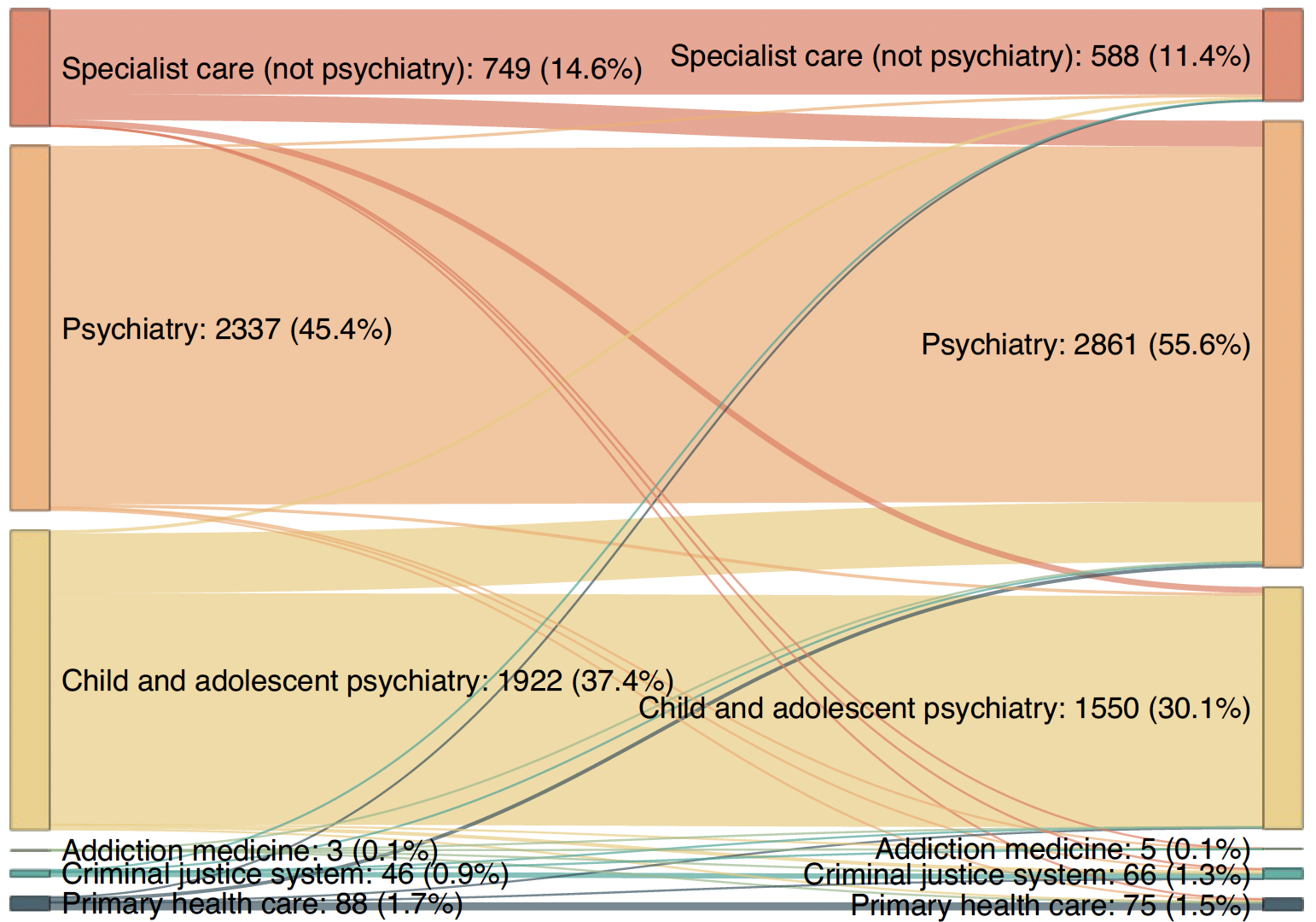


# **eFigure 5.** Inclusion overview for sensitivity analysis including only individuals with substance use disorder defined as any ICD 9 or 10 alcohol or substance related diagnosis.

33,990 controls without SUD

248 emigrated before SUD Dx

13 last Rx before SUD is single Rx

1 without matched control

275,549 individuals who have ADHD diagnosis (1986-2020)

229,368 individuals dispensed ADHD medications

195,941 individuals **free of SUD** at the time of first ADHD diagnosis or medication dispensation

7,061 individuals **on ADHD** treatment^a^

6,799 eligible cases with SUD

Controls matched 5:1 to cases

3,091 (45.5%) discontinued ADHD^b^ medication^b^

3,708 continued ADHD medication^b^

1,165 (37.7%) reinitiated ADHD medication^c^

1,926 did not reinitiate ADHD medication^c^

16,159 individuals with SUD diagnosis

8,588 (25.3%) discontinued ADHD medication^b^

25,402 continued ADHD medication^b^

3,711 (43.2%) resumed ADHD medication^c^

4,877 did not resume ADHD medication^c^

Dx: diagnosis; Rx: medication prescription

^a^ defined by SUD Dx covered by ADHD treatment (ADHD continuous treatment defined using 90-day grace period)

^b^ within 1 year from date of SUD diagnosis or corresponding time

^c^ within 1 year from date of ADHD medication discontinuation
